# Editorial: “AAV Gene Therapy: Immunology and Immunotherapeutics”

**DOI:** 10.3389/fimmu.2021.822389

**Published:** 2021-12-21

**Authors:** José M. Martinez-Navio, Nicole K. Paulk, Guangping Gao

**Affiliations:** ^1^ Department of Pathology, Miller School of Medicine, University of Miami, Miami, FL, United States; ^2^ Department of Biochemistry & Biophysics, University of California San Francisco, San Francisco, CA, United States; ^3^ Horae Gene Therapy Center, University of Massachusetts Medical School, Worcester, MA, United States; ^4^ Li Weibo Institute for Rare Diseases Research, University of Massachusetts Medical School, Worcester, MA, United States; ^5^ Department of Microbiology and Physiological Systems, University of Massachusetts Medical School, Worcester, MA, United States

**Keywords:** AAV, gene therapy, immunogenicity, capsid antibodies, cellular responses, innate immune activation, immunotherapeutics

Adeno-associated virus (AAV) has become the vector of choice for current gene therapy approaches. AAV is a small, single-stranded DNA virus which effectively infects humans and other vertebrates without causing disease. AAV is highly infectious but naturally replication-defective in the absence of a helper virus, and its genome is simple to manipulate. To generate a recombinant AAV (rAAV) vector, the viral genes are replaced with a transgene expression cassette, while the flanking inverted terminal repeats (ITRs) required for encapsidation, are retained. Virion capsid proteins for encapsidation of the vector DNA are provided in *trans* and the resultant rAAV is subsequently purified. The safety profile of rAAV vectors is well established from decades of research and over 200 clinical trials to date. Many more are forthcoming with numerous investigational new drug applications in various stages of review. AAV-mediated gene transfer has benefited numerous individuals with genetic diseases by mediating long-term expression of the transgene. However, some hurdles remain, such as pre-existing immunity to the rAAV capsids and unwanted immune responses to the transgene product. A total of 13 articles in this Research Topic examine current immunological barriers in the field of rAAV-based gene therapy and immunotherapeutics, evidencing the latest discoveries on approaches on how best to overcome them.

Pre-existing neutralizing antibodies to the AAV capsid are found in a significant percentage of the human population from exposure to circulating wild-type AAV. These antibodies substantially reduce the transduction efficiency of rAAV and can prevent successful delivery of the transgene in those individuals. In this Research Topic, Weber reviews this topic, including descriptions of what the prevalence of such antibodies are in the general population, and the difficulties associated with measuring these antibodies in a way that is predictive of therapeutic outcomes. Importantly, the author also discusses potential solutions to these issues. In line with this, an approach that holds great promise to overcome natural pre-existing immunity is the rational design of engineered AAV capsids. In a perspective article, Wec et al. discussed how machine learning and high-throughput screening can expand the landscape of engineered capsids, which can make AAV therapies safer and more broadly applicable.

While rAAV vectors have been traditionally seen as non-immunogenic, immune responses can be generated to the AAV itself and/or to the transgene product. In a perspective article, Hamilton and Wright discuss the inherent immunogenicity of the rAAV vectors, and how they can trigger both innate and adaptive immune responses (the latter including both cellular and humoral responses) and mediate complement activation. Innate immune responses can be triggered by the rAAV capsid, but also by the vector’s DNA genome, which can trigger toll-like receptor 9 (TLR9) activation. TLR9 recognizes unmethylated CpG motifs (cytosine guanine dinucleotides) and is expressed in different sets of immune cells, including dendritic cells, macrophages, and other antigen presenting cells. Activation of TLR9 triggers the secretion of pro-inflammatory cytokines and the recruitment and activation of cytotoxic T-cells which can, in turn, mediate elimination of transduced cells. Bertolini et al. have reported a deimmunization strategy that consists of removing these CpG motifs from the vectors. In a rodent model of hemophilia B, the authors have shown markedly reduced cytotoxic T-cell infiltration after intramuscular administration of CpG-depleted rAAV. Importantly, this approach resulted in improved preservation of transduced cells. Immune-mediated rejection and clearance of rAAV-transduced cells is a crucial issue during gene therapy. In a review focused on cellular responses, Ertl has discussed how CD8 T-cells can recognize and destroy rAAV-transduced cells. Notably, ITRs flaking the transgene cassette in rAAV can be sensed by DNA damage response proteins in transduced cells. Dudek and Porteus have reviewed the current understanding of DNA damage response and innate immune activation to both genomes and capsids during early steps of vector transduction. As reviewed by Chu and Ng, current strategies to prevent or ameliorate unwanted host immune responses during gene therapy include an array of potential pharmacological immunosuppressive and immunomodulatory regimens.

Post-translational modifications were recently discovered on rAAV capsids and could be contributing to the undesired immunogenicity seen in some gene-therapy recipients. Recent concerns regarding immunogenic responses to high-dose intravenous administration of rAAV has highlighted the need for extensive analysis of the preparations in order to identify and catalog potentially immunogenic vector lot components. In this Research Topic, Rumachik et al. have described a mass spectrometry workflow to thoroughly analyze and characterize post-translational modifications in rAAV capsids and on potential host cell protein impurities carried over in the vector preparation. Extensive rAAV characterization promotes enhanced batch-to-batch consistency and is crucial for the development of safer and more reliable rAAV vectors.

Viral tropism, or the ability to infect a specific tissue or cell type, is a key factor to consider when selecting the most appropriate rAAV serotype for gene delivery. Viral tropism is largely determined by the rAAV capsid, and a variety of different capsid serotypes have been identified. When rAAV vectors are produced in the laboratory, an AAV2 ITR genome is engineered to contain the transgene of interest and the resulting recombinant genome is encapsidated in the preferred capsid. Depending on the desired target cells or tissues, different serotypes may be preferred. Brown et al. have developed an experimental and computational approach based on single-cell RNA sequencing to characterize *in vivo* viral tropisms and uncover targeting biases. An additional potential source for immunogenicity against the rAAV-expressed transgene product is related to off-target delivery. Inadvertent transduction of antigen presenting cells (APCs) can result in presentation to the immune system, and trigger an immune response. To prevent this, Muhuri et al. have used miRNA biding sites to efficiently block transgene expression in APCs. miRNAs are small non-coding RNAs that function in RNA silencing. By using miRNA binding sites specific for miRNAs found in APCs, but not in target cells, expression can be carefully ablated in APCs while sparing target tissues. rAAV vectors containing this detargeting strategy strongly inhibited cytotoxic T-cell activation and the generation of antibodies against the transgene. This approach mediated higher levels of transgene expression *in vivo*.

Although mostly used to treat monogenic diseases, gene-transfer mediated by rAAV can also be exploited to deliver immunotherapeutics, such as monoclonal antibodies. The coding sequence of properly characterized protective/neutralizing antibodies against a pathogen of interest can be delivered *via* rAAV, thus aiming to prevent or treat infectious diseases and confer long-lasting immunity. This strategy passively bypasses the immune system as no immune response to an immunogen is required. Zhan et al. have reviewed this topic, highlighting how rAAVs can become game changers in our fight against transmissible diseases, including HIV, dengue, influenza and others. Interestingly, antigens of interest can also be delivered in rAAV vectors with the aim of conferring protection to a disease in the recipient. Shahnaij et al. have reported a vectored vaccination regimen against malaria that conferred full protection to malaria-parasite challenge in a rodent model. The vaccination regimen consisted of a prime with human adenovirus and a booster inoculation with AAV8, both encoding the *Plasmodium falciparum* circumsporozoite protein (a protein of the sporozoite’s surface).

As depicted by Rapti and Grimm in their review article, there is a continuous immunological and molecular race between AAV and its human host. Current and upcoming advances in rAAV gene therapy as described in the compelling articles of this Research Topic, promise safer and more broadly applicable therapies in the future. We invite you to read each of these enlightening papers and reflect on the Research Topic as a whole.

## Author Contributions

All authors listed have made a substantial, direct, and intellectual contribution to the work and approved it for publication.

## Conflict of Interest

GG is a scientific co-founder of Voyager Therapeutics, Adrenas Therapeutics and Aspa Therapeutics and holds equity in the companies. NP and GG are inventors on patents related to AAV-based gene therapies, some of which have been licensed to commercial entities.

The remaining author declares that the research was conducted in the absence of any commercial or financial relationships that could be construed as a potential conflict of interest.

## Publisher’s Note

All claims expressed in this article are solely those of the authors and do not necessarily represent those of their affiliated organizations, or those of the publisher, the editors and the reviewers. Any product that may be evaluated in this article, or claim that may be made by its manufacturer, is not guaranteed or endorsed by the publisher.

